# How do family carers and care-home staff manage refusals when assisting a person with advanced dementia with their personal care?

**DOI:** 10.1177/14713012221123578

**Published:** 2022-09-02

**Authors:** Tamara Backhouse, Yun-Hee Jeon, Anne Killett, Eneida Mioshi

**Affiliations:** School of Health Sciences, 83726 University of East Anglia, Norwich, Norfolk, UK; Sydney Nursing School, 4334 The University of Sydney, Sydney, NSW, Australia; School of Health Sciences, 83726 University of East Anglia, Norwich, Norfolk, UK

**Keywords:** resistance-to-care, rejection, activities of daily living, social care

## Abstract

**Background and objectives:**

Caregivers may encounter, or inadvertently cause, refusals of care by a care recipient. Managing refusals of care can be challenging and have potential negative consequences. We aimed to examine caregivers’ (care-home staff and family carers) experiences of managing refusals of personal care in advanced dementia.

**Research design and methods:**

One-to-one semi-structured interviews with 12 care assistants from six care homes and 20 family carers who were physically assisting a person with advanced dementia with their personal care in the UK. Interviews were audio recorded and transcribed verbatim, with data analysed using qualitative content analysis.

**Findings:**

Core to the caregiver experience of refusals of care was ***knowing the person***. This underpinned five key themes identified as caregivers’ strategies used in preventing or managing refusals of care: (1) finding the right moment to care; (2) using specific communication strategies; (3) being tactful: simplifying, leaving, or adapting care; (4) having confidence in care; and (5) seeking support from others when safety is at risk.

**Discussion and implications:**

Different caregiver relationships with the person with dementia influenced how they managed refusals of care. Refusals of care can place caregivers in tough situations with tensions between providing care when it is seemingly not wanted and leaving care incomplete. Both caregiver groups require support such as coaching, mentoring and/or advice from other health and social care practitioners to manage difficult personal care interactions before crisis points occur.

## Background

People living with advanced dementia often require assistance with their personal care (Prizer & Zimmerman, 2018). This assistance can be defined as any physical support given to the person to conduct basic activities of daily living such as eating, bathing, going to the toilet, dressing, or oral care (Health and Social Care Act, 2008). Dementia is a progressive neurological condition with a range of cognitive and behavioural symptoms. Consequently, people with dementia often require more assistance with personal care than older adults without dementia (Prince et al., 2015).

Behaviours viewed as related to dementia can be framed and termed in different ways, for example, what may be termed as aggression or agitation by professionals may be viewed as anger or frustration by people with dementia (Burley et al., 2021). There may be similar discrepancies around the term ‘refusal of care’, which implies a deliberate act from the person with dementia. In alternative framings, refusals of care, also termed resistiveness-to care/rejection of care (Galik et al., 2017; Ishii et al., 2012), have been defined as meaningful communicative actions by the care recipient invoked from the care interaction (Spigelmyer et al., 2018a; Volicer, 2020).

Actions perceived as refusals can include the care recipient stiffening their body, being verbally or physically aggressive, or gripping onto things (Mahoney et al., 1999; Volicer & Hurley, 2015). They can be the result of family carers or care-home staff (collectively termed as caregivers hereafter) not taking the time and using an acceptable approach to engage with the person with dementia (Ishii et al., 2012). Therefore, the person with dementia may not understand the caregiver’s intentions (Galindo-Garre et al., 2015), resulting in either intentional or reactional refusal of care. Factors contributing to refusals include caregiver touch, the verbal interaction, personal care provision in general (Belzil & Vezina, 2015; Ishii et al., 2012), and the person with dementia experiencing depression (Galindo-Garre et al., 2015), pain or psychotic symptoms such as delusions and hallucinations (Galindo-Garre et al., 2015; Ishii et al., 2010).

In the UK, 58% of people with dementia are in the advanced stages (Wittenberg et al., 2019), when refusals are most common (Ishii et al., 2012). The advanced stages are characterised by increasing difficulties communicating; being largely unaware of recent experiences, places, dates, or events; personality and emotional changes, and a requirement for assistance with activities of daily living such as washing, dressing, and going to the toilet (Reisberg et al, 1982). Refusals can be distressing for the person with dementia and their caregiver, they can create difficulties in care interactions, and exacerbate caregiver burden (Cheng, 2017; Choi et al., 2019; Fauth et al., 2016; Ishii et al., 2012; Spigelmyer & Schreiber, 2019). All people have the right to refuse care, including people with dementia (Social Care Institute for Excellence, 2021). However, if care is repeatedly refused negative consequences could arise such as poor hygiene or infections, which the person with dementia may not understand (Backhouse, 2021). Practice guidance advises that learning and attending to a person’s reasons for refusing care is paramount (Social Care Institute for Excellence, 2021).

Playing recorded music during care and using different bathing techniques such as, strip washes and thermal baths can reduce refusals of care in dementia (Backhouse et al., 2020; Konno et al., 2014). These approaches aim to make the care experience more acceptable to the person. This aligns with person-centred care (Kitwood, 1997), which promotes valuing the person living with dementia, identifying the person’s perspectives, offering individualised care, and producing reassuring social environments (Brooker & Latham, 2016). All of these, if adopted, may potentially enable caregivers to relate better with the person with dementia, so that the caregiver’s intentions are understood and acceptable.

Refusals occur in both family (Fauth et al., 2016) and care-home settings (Konno et al., 2014) where contexts and the nature of relationships are different and likely to be central to the management of refusals. Family carers may have better insight into the person’s preferences, patterns, and behaviours, informing strategies personally attuned to the individual with dementia, but may also experience more emotional consequences of negative care interactions including refusal of care (Schulz & Eden, 2016). In contrast, care staff in care-home settings must cope with time and workload pressures (Duan et al., 2020). They, and the nature of their work, are typically undervalued by their employing organisations, potentially influencing their confidence and the care they provide (Kadri et al., 2018). However, care staff have usually received dementia training covering interacting with people with dementia (Smith et al., 2019) and are experienced in assisting with personal care.

Recent qualitative studies have shown that both care staff and family caregivers find refusals uncomfortable and challenging, with potential to contribute to psychological distress and burden (Mortensen et al., 2021; Rathnayake et al., 2019). When facing refusals, family carers can question their own ability to care, start to perceive themselves as more ‘carer’ than a spouse or child, and show surprise at their own reactive behaviours (Spigelmyer, Hupcey, Smith, et al., 2018). In contrast, care staff can feel caught between completing care activities and upholding the person’s autonomy (Mortensen et al., 2021). However, the strategies caregivers employ to manage refusals of care day-to-day are not well known.

Using a standpoint of the perspectives of caregivers, the aim of this research was to examine how family carers and care-home staff in England experience and manage refusals of assistance with personal care in advanced dementia.

## Methods

This qualitative study employed semi-structured interviews and inductive content analysis. As with much qualitative research (Sandelowski, 2010), our study emphasises gaining understanding of an experience, while not being explicitly guided by an established collection of philosophic assumptions (Caelli et al., 2003).

### Participants

**Figure 1.**
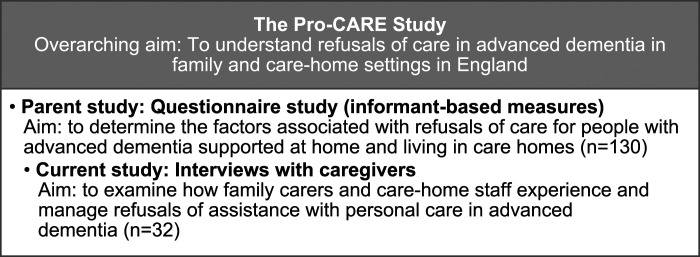
Pro-care study design.

This current study was linked to a parent study [Pro-CARE], funded by the Alzheimer’s Society, conducted in England, examining refusals of care in advanced dementia. Figure 1 shows the overall research design. All participants in the current study had previously taken part in the parent study. Participants were care-home staff members supporting a resident and family carers supporting a person living at home. All care recipients had advanced dementia and were aged 65 or above. These two caregiver groups were chosen to enable learning from each distinct setting to contribute to the development of comprehensive educational materials for caregivers of people with dementia. Dementia severity was assessed as part of eligibility for the parent study using the Frontier Dementia Rating Scale (FRS), which is a well-validated, informant-based, staging tool incorporating aspects such as behaviour, self-care, and household chores (Mioshi et al., 2010). Dementia sub-type was determined from General Practitioners as part of the parent study.

### Recruitment to the study

Both care-home staff and family carers taking part in the parent study, living in the East of England could opt-in to take part in a qualitative interview on the consent form (relating to both studies). Care-home staff were recruited from six care homes identified from a directory in the public domain as providing specialist dementia care in the East of England, where the care-home managers, after an invitation letter, telephone call and meeting with the first author, had agreed for their homes to be involved in the study. Care homes included in the study were typical care homes in England providing accommodation, food and 24-h care, including assistance with personal care. They were either nursing homes providing qualified nursing care (n = 1) or residential homes providing care without qualified nursing (n = 5). Care-home staff in this study included care workers (not managers or registered nurses) who provided physical assistance with personal care to the person with dementia at least six times per week, had conversational English, and were aged 18 or over. Managers provided study information to care staff and residents/residents’ family members they thought would be eligible and interested. For family carers, two recruitment strategies were used: 1) dissemination of information leaflets through community dementia services in the East of England; and 2) accessing local and national research registers. Invitations were sent to those family carers who had previously registered their interest in participating in dementia research either in a local East of England research team database or in a national digital database for dementia research, called Join Dementia Research. Family carers were included if they lived in the East of England, were the primary carer (family member or close friend) for the person with dementia, provided physical assistance with personal care, had conversational English, and were aged 18 or over.

From those consenting to take part in an interview (n = 20 care-home staff and n = 30 family carers), maximum variation purposive sampling (Etikan et al., 2016) was conducted to collect data from n = 32 caregivers (n = 12 care-home staff and n = 20 family carers) using information collected in the parent study to ensure a range of experiences of providing personal care. For example, we sought caregivers experiencing several refusals of care and those experiencing only a few, and caregivers assisting the person with dementia with all personal care activities and those assisting with only select care activities such as showering and going to the toilet. Male and female caregivers and caregivers with different relationships to the care recipient were also sought.

### Study procedures

#### Ethics and consenting procedures

Ethical approval was sought from, and a favourable opinion provided, by the Queen’s Square Research Ethics Committee, London [Reference: 251339].

Written consent was obtained for all participants, including people with dementia for the parent study who had questions asked about them and their dementia subtype determined from their General Practitioners. Capacity to consent was assessed by the first author through talking to the person with dementia about the research and using a guide sheet to document whether the person could understand the research and consequences of taking part or refusing, retain and weigh up the information in relation to the research decision, and communicate their decision at that time. In-line with the Mental Capacity Act (2005), if a person with dementia lacked the capacity to consent, a relative or close friend acted as a consultee: they were provided with study information and advised the researcher if they thought the person would have been likely to participate if they had capacity to make the decision. Assent was sought from people with dementia when appropriate.

#### Data collection

Two family carers and one care-home staff member who assisted people with advanced dementia with their personal care had research adviser roles on the study as ‘experts by experience’. They regularly provided advice and insights from their own experiences to the first author throughout the study. These advisors assisted the first author to develop the interview topic guide (see Online Supplementary Material). Advisors suggested aspects they viewed as relevant to personal care assistance for people advanced with dementia. The topic guide was then developed by the first author drawing on their own past experiences as a care-home worker and researcher of dementia care and the advisors’ ideas. The advisors and AK then reviewed and agreed the topic guide. Interview questions focused on three broad areas (1) ‘supporting the person with dementia with personal care’ to examine usual care situations for the dyad, (2) ‘strategies used to provide care’ to examine how caregivers managed refusals of care, and (3) ‘available support’ to examine caregiver sources of support to assist with refusals of care. Semi-structured, face-to-face interviews with caregivers took place in 2019 and early 2020 before the COVID-19 pandemic. Interviews were recorded and transcribed verbatim. Interviews took place in family carers’ homes and at care homes. The first author conducted the interviews; they are an experienced care-home care worker and post-doctoral research fellow proficient in qualitative research in dementia care. Data collection was stopped when only similar actions and no new caregiver strategies to manage refusals were being reported from each setting (Saunders et al., 2018).

#### Data analysis

Inductive qualitative content analysis was undertaken (Elo & Kyngas, 2008). This approach involves open coding, creating categories and using abstraction to generate generic categories. The focus of the analysis was on the way caregivers coped with perceived refusals of care from the care recipient. The unit of analysis was each caregiver.

**Open coding:** First, the first author engaged with all transcripts and became familiar with the data. The three ‘expert by experience’ research advisors, who were trained in the coding process engaged with a subset of transcripts. They discussed their interpretations and thoughts about what these data meant with the first author who wrote notes. The first author then read the notes and all transcripts, formulated meanings about the content, and generated tentative codes. Once, tentative codes were developed they were refined as a team with all authors via multiple discussions and iterations. A few codes were distinct to family or care-home settings, but most were common to both participant groups. All data were then coded applying the final agreed codes using NVivo12 pro (QSR 20.4.0.4) to manage the process, with the final few transcripts contributing only similar information about caregivers’ actions and management strategies indicating data saturation was achieved (Saunders et al., 2018).

**Figure 2.**
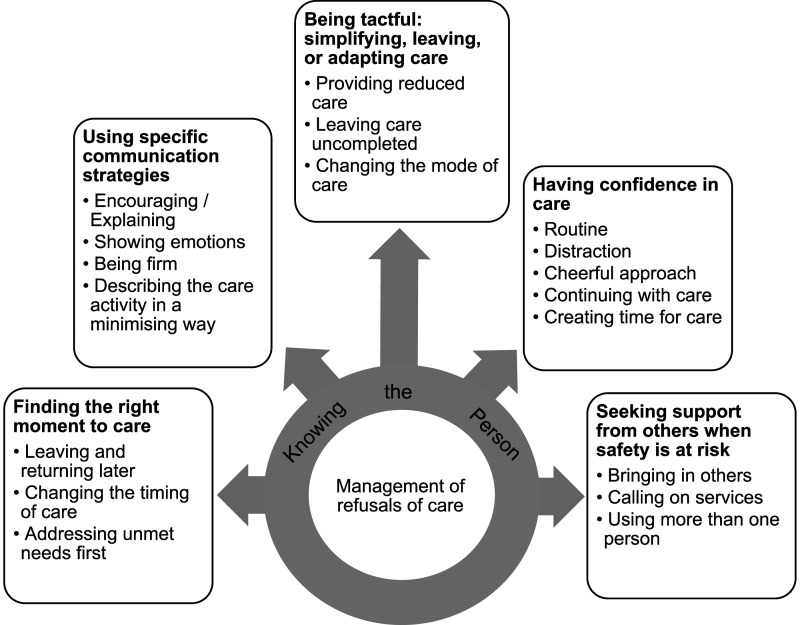
Analysis main category “management of refusals of care” linked to five generic categories, and further subcategories.

**Creating categories:** Codes were examined, with vital qualities compared and grouped with related observations into higher level categories (sub-themes) to which they belonged such as the category ‘leaving and returning later’ (see Figure 2).

**Abstraction**: Categories were then examined and abstracted to generate higher generic categories (themes). This stage involved grouping categories (sub-themes) with similar events and incidents together (Elo & Kyngas, 2008) to produce generic categories such as ‘finding the right moment to care’ that adequately reflected the research findings.

During the creating categories and abstraction stages meanings, names and interpretations of categories (sub-themes) and generic categories (themes) were discussed multiple times, refined and agreed with all authors. Ongoing team discussions during the data analysis process helped ensure credibility of the findings.

## Findings

Table 1 shows the characteristics of the sample. We conducted 32 interviews with 20 family carers and 12 care-home staff from six different care homes. Twenty-eight caregivers were female (87.5%), average age was 61 and all were White British. Most family carers were spouses (65%) and care-home staff, care assistants (83%). Family carers had been assisting the person with dementia an average of 5-years and care-home staff 2-years. Fifty percent of people with dementia receiving assistance with personal care were female, Alzheimer’s Disease was the most common sub-type of dementia (30%), and most were in the severe stage of dementia (69%). Personal care assistance required by the care recipients included help with washing/showering, dressing, going to the toilet, catheter care, eating, oral hygiene, and hair and nail care.

**Table 1.** Descriptive characteristics of sample.

	Total sample (*n* = 32)	Family carers (*n* = 20)	Care-home staff (*n* = 12)
Female % (*n*)	87.5 (28)	85 (17)	92 (11)
Age average (range)	61 (19–79)	71 (55–79)	47 (19–65)
Ethnicity % (*n*)			
White British	100 (32)	100 (20)	100 (12)
Length of time caring for care recipient years average (range)	4 (0.5–10)	5 (2–10)	2 (0.5–7)
Care recipient female % (*n*)	50 (16)	40 (8)	66.6 (8)
Care recipient type of dementia % (*n*)			
Alzheimer’s disease	31 (10)	30 (6)	33 (4)
Vascular	19 (6)	20 (4)	17 (2)
Mixed	16 (5)	10 (2)	25 (3)
Frontotemporal	9 (3)	15 (3)	0 (0)
Parkinson’s	3 (1)	5 (1)	0 (0)
Unknown	22 (7)	20 (4)	25 (3)
Care recipient severity of dementia % (*n*)			
Severe	69 (22)	70 (14)	67 (8)
Very severe	28 (9)	25 (5)	33 (4)
Profound	3 (1)	5 (1)	0 (0)
Family carer relationship to person with dementia % (*n*)			
Spouse		65 (13)	
Adult child		20 (4)	
Adult child-in-law		5 (1)	
Close friend		10 (2)	
Care-home staff role % (*n*)			
Senior care assistant			17 (2)
Care assistant			83 (10)
Interview length minutes average (range)	50 (25–78)	57 (40–78)	38 (25–49)

Figure 2 shows five analysis categories, all underpinned by ***knowing the person***: (1) finding the right moment to care; (2) using specific communication strategies; (3) being tactful: simplifying, leaving, or adapting care; (4) having confidence in care; and (5) seeking support from others when safety is at risk. ***Knowing the person*** refers to caregivers’ ability to understand and respect the person’s mood, ability, preferences, needs and perspectives, which was crucial in their delivery of tailored care to the person.

### Finding the right moment to care

Finding the right moment to care was a priority for both caregiver groups. Often caregivers ascertained the person’s mood before attempting care and withdrew if they perceived the person as unreceptive at that time:

*… he was wet in his chair and we really felt that we needed to get him changed, however, there was no way he was going to accept that without extreme violence so in that case it was just best to leave him … yes, he was wet, and we were all upset that he was wet … later it was fine* (Care Home 2, Care Assistant 3)

Here, even though the personal care was considered essential and uncompleted care could have reflected negatively on the care home’s reputation if noticed by visitors to the home, a delay enabled a better care interaction. Delaying care demonstrated caregivers’ understanding of the person’s disposition at that time and that it was interpreted as transitory.

The timing of care could reduce the likelihood of refusals occurring. Family carers reported learning to time their care interventions carefully:

*If she falls asleep in the evening … and I leave her too long and she goes into a deep sleep … then there is a real problem … she’s woken up and she doesn’t know where she is, so … getting her clothes off, or her pyjamas is difficult if she shouts at you … I’ve realised I mustn’t let her do that, got to get in there and do things before* (Family Carer 8, Daughter)

Prior experiences of the person’s daily pattern meant care was brought forward, showing that the family carer perceived the evening sleep contributed to refusals and that this could be circumvented. By timing care well, caregivers could create more acceptable care interactions for the person and fulfil their own agenda to complete care. Identifying and addressing unmet needs before offering care was also described, demonstrating caregivers’ awareness of care recipients’ experiences and changing aptitudes for receiving care.

### Using specific communication strategies

Communication strategies could be used to smooth care processes or check the person’s actions. Both caregiver groups reported that explaining what they were doing and why, or encouraging the person could work to side-step refusals of care:

*I stepped back [laughs]. I didn’t want to get thrown on the floor and I would ... I’d say “listen, I’m just helping you get clean, we’ve got to get you clean before I can get you back into bed”* (Family Carer 40, Spouse)

This family carer shows an understanding that if the person can grasp what she is trying to do, and why, they will be more likely to agree to the care.

Some family carers and care-home staff reported that if refusals were aggressive, they would be firm with the person:

*… if it’s the fist type of aggression then I’ll give the warning “don’t you dare” and it usually works, usually.* (Family Carer 15, Spouse)

*… if he gets really aggressive … I don’t use it very often, but I will say to him “now come on, you’re a man, I’m a woman, you don’t hit women” … it will stop him dead* [completely] (Care Home 2, Care Assistant 3)

Knowing the person enabled caregivers to judge whether being firm was appropriate. Warnings had the effect of checking the person’s aggressive behaviour during care; an important factor, since some caregivers were fearful of the person they were supporting. Of note, the family carer’s language is more direct than that of the care staff’s, reflecting the context of the familial relationship. The words ‘don’t you dare’ may be deemed acceptable within a family interaction but unprofessional in a care home context.

A few family carers reported that they found refusals very difficult and would show their emotions:

*I almost feel tearful sometimes and when he’s got me to that pitch, he realises. … I say “why do you have to go through this every time? What do you think it does to me?” … I’m showing my frustration* (Family Carer 24, Spouse)

The emotion and intimacy shown reflects the close and long-term relationship context of family settings. The emotional toll on many family carers was considerable, echoing their long personal journey adapting to dementia, enduring caring responsibilities, with refusals becoming part of their personal relationship.

Care-home staff reported describing the care activity in a minimising way to try to persuade the person to go ahead:

*… someone who refused to go to the toilet ... “look, I just need to check your pad and of course once when we’ve done that, you can come and sit down back here, you can have a cup of tea”* (Care Home 6, Care Assistant 2)

Use of the words ‘just’ or ‘quick’ and pointing out the benefit of the activity were often reported by care-home staff demonstrating an understanding that the person may feel overwhelmed or put off by the thought of the care activity. The care home context allowed an emotional distance and framing of the refusal as transactional, contrary to the intensity of the close personal relationships in family settings.

### Being tactful: Simplifying, leaving or adapting care

Sometimes working around refusals was not possible, or the thought of provoking a refusal or upsetting the person further was not deemed acceptable, so both caregiver groups left perceived optimal care uncompleted:

*I mean there were occasions when staff wouldn’t shave him, couldn’t, … they felt they couldn’t attempt it …* [the family] *said “without distressing him just leave him … if he grows a beard it is better than upsetting him”* (Care Home 1, Care Assistant 3)

*… where I think, well I’m not going to win this battle, I just let him carry on. He’ll win that battle by keeping his pyjamas on or putting his jumper on back to front* (Family Carer 4, Spouse)

In these instances, caregivers asked themselves if it mattered if care was different or incomplete. Reduced care was viewed as tolerable, particularly if the care required was not desperate/essential. Some family carers framed issues with completing personal care as ‘battles’ showing they regarded refusals as personal encounters, whereas care-home staff were able to view refusals from a more distanced perspective, withdrawing and seeking advice from other staff and family members.

Adapting modes of care was often used to prevent or overcome refusals in both settings, often relying on understandings of the person’s preferences:

*… she doesn’t like the showers … she definitely doesn’t want, so I wouldn’t push her … I don’t think it’s fair on her and I don’t think it’s necessary because I can give her a good* [strip] *wash and probably check her better as well* (Care Home 5, Senior Care Assistant 2)

Adapting the mode of care was part of a problem-solving approach, which enabled adequate care, but in a way more acceptable to the person with dementia. Caregivers used their knowledge of the person to find mutually acceptable compromises. Moving to strip washes rather than baths or showers, no longer shaving, using dry shampoo, and bed days were all employed.

### Having confidence in care

Most caregivers reported using approaches that were acceptable to the person with dementia. Care-home staff understood knowing the person well facilitated the use of a familiar routine and enabled distraction by talking about the person’s interests to enhance their receptiveness to care:

*I need* [to] *know the history of* [the person with dementia] *… know what he likes, what he likes to talk about … you learn that this is how to speak to him* (Care Home 1, Care Assistant 1)

They talked of approaching the person with a cheerful attitude:

*… she can be a little bit challenging sometimes but if you go in with the right sort of attitude with her, she does tend to bend a little bit* (Care Home 2, Care Assistant 4)

Care-home staff demonstrated a sense of self-efficacy; they appeared to have confidence that it was within their power to turn a potential refusal situation around.

A small number of family carers just carried on with care through the person’s protestations:

*I don’t listen to the ‘complain’ unless it becomes really forceful and then I have to … otherwise I, I just couldn’t control it, you can’t go back, when she is already dressed … to go backwards and undress that is much more difficult than actually … doing it from the start* (Family Carer 8, Daughter)

This family carer took control as a way to cope with not knowing if care activities were completed properly by the person with dementia. This meant they overruled the person’s attempts to oppose care, potentially escalating or prolonging a refusal. At other times, family carers would carry on with the care activity to complete a feared care task after repeatedly putting it off, knowing from past experiences that the person would not like it:

*… cut cut cut and it’s done very quickly, if I don’t challenge what she’s saying to me I just “put your hands in water, right let me cut” “No I don’t want them, oh no you are hurting me” “Right that’s one hand done give me the other hand!”* (Family Carer 9, Daughter)

The family carer was determined to complete the nail cutting activity and do it as quickly as possible to reduce potential distress. Carrying on with care while a person is refusing may not be optimal care. However, family carers who had sole responsibility for assisting with care activities may have felt pressure to complete tasks.

### Seeking support from others when safety is at risk

Support and safety were different for family carers and care-home staff. Family carers relied on relatives and neighbours for advice and support:

*I get to a stage where I can’t cope with him because he might be yelling and shouting and I can’t do anything, my neighbour … I give her a ring and if she’s in, she’ll come in here … he’ll stop yelling straight away because it’s someone outside the home* (Family Carer 4, Spouse)

This carer perceives herself as having limited capability to manage this type of refusal situation on her own. Knowing the person’s usual response when other people were present offered this carer a way forward. If family carers could not manage, they often relied on paid homecare services. In one case where a family carer had no immediate support, they called the Police:

*That was the first time that I have felt the fear … if he* [husband living with dementia] *was successful in realising that I was afraid it would become very very difficult, so I think it* [calling the Police] *was the best way to go. ... she* [the police person] *has referred us to the Elderly Intensive Support Group* (Family Carer 15, Spouse)

Here, the family carer understood the person and the consequences for their relationship if she showed fear, therefore she implemented robust action to prevent this.

If care-home staff found providing personal care difficult, they relied on the staff team around them for their support:

*If one of us can’t get somebody up* [out of bed and washed and dressed]*, then they will try and find somebody else. … with that lady that I was saying about in the shower and gets a bit agitated, normally they come and find me ... I’m actually her key worker* [a staff member with a close relationship with the resident, who liaises with their relatives/friends and ensures personal requirements are met] (Care Home 3, Senior Care Assistant 2)

The care home context allowed this swapping of care staff, often to those staff knowing the person well, demonstrating another transactional aspect of care. Care-home staff could also use more than one person to conduct care:

… *it usually takes two of us … only one of us does the talking because if we are both going ‘try and stand up [name], can you move your foot here, can you do that’ then he can’t cope with that … we usually play … one good cop and one quiet cop* (Care Home 2, Care Assistant 3)

Using ‘double ups’ often assisted with the management of refusals of care but also made assisting the person to move safe for example when using a hoist in accordance with staff training. Knowledge of the person enabled staff to circumvent overwhelming them with too much communication.

## Discussion

This is a novel study examining how family carers and care-home staff managed refusals of care for people with advanced dementia on a day-to-day basis. Knowing the person was vital and underpinned all strategies, since caregivers could better assess the person’s mood and tailor care to individual preferences and needs. Caregivers worked flexibly to make care more acceptable to the person with dementia and prevent refusals occurring. Importantly, both caregiver groups generally used the same key strategies when managing refusals of care, despite differences in setting and years of caring experience. However, there were differences in the meaning of the strategies in terms of the caregiver contexts, and nature and quality of their relationships. Family carers were personally invested, living within the personal relationship consequently, their emotional investment was prominent. Care-home staff were more detached, they frequently consulted others and care could be more transactional.

Although some family carers in our sample sought and received support from family members or home-care workers, they had less immediate support to manage refusals than care-home staff and found refusals of care difficult. Many felt emotional strain, with key events or a cumulative effect over time leading a few to reach crisis point. This corresponds with previous research showing heightened burden in family carers assisting people with advanced dementia with their care, particularly with those exhibiting behaviours seen as aggressive (Cheng, 2017; Haro et al., 2014). Family carers’ internal position within the situation meant some of them framed refusals of care as ‘battles’ and talked of the person with dementia ‘winning’. Being the only person with the responsibility to care for the person with dementia over long periods of time made two family carers in our sample resort to completing care regardless of any refusals. Of note, in these instances care recipients were female. Non-consensual care has been identified before but examined from mainly professional caregiver experiences, showing its use as a last resort (Backhouse et al., 2018), guiding staff who find it necessary to resort to restraint use in extreme circumstances to act proportionately (Sells & Howarth, 2014) and considering the role of clinical psychologists in supporting those encountering difficult care situations (Watts et al., 2017). In our data, motivation to continue with care when a person with dementia was refusing was to maintain control of the routine or to complete specific, feared, and unresolved care tasks.

Due to the policy drive to keep people in their own homes (Carter, 2016) and the financial resources family carers save the state (Wittenberg et al., 2019), preparing and supporting family carers to manage refusals and continue in their role, if they wish to, is key. Such support could involve ensuring timely availability of key external resources, providing information and advice and/or carer training, coaching, or mentoring, (Parkinson et al., 2017) or respite from personal care activities (Chung-Ying et al., 2019).

Care-home staff meeting the person with dementia when they were already significantly cognitively impaired meant staff often relied on family member information about the person and developing a rapport with the person over time. Despite care-home staff developing close relationships with residents, the comparative distance compared to family carer relationships allowed staff to be emotionally less affected by refusals of care. There were still times when emotional labour was evident (Johnson, 2015), such as when staff needed extra support; they found refusals difficult and frustrating to manage, or when they were fearful of being hurt by the person refusing.

Providing person-centred care in institutions involves a knowledge of the person and their history, individual respect for the person, collaboration in care, and an approach where all staff levels and roles are involved (Hunter et al., 2016; Killett et al., 2016; Stein-Parbury et al., 2012). In our study, the organisational culture and a shared purpose in providing care facilitated care-home staffs’ management of refusals through a team effort by enabling staff to swap or provide care in twos (Killett et al., 2016). Typically, family carers have less options for teamwork, since family members, friends, or home-care workers are not always available. Care-home staff’s communication strategies such as using the word ‘just’ have been found previously to work in acute hospital settings to convey a sense that an action will be quick and unproblematic (O’Brien et al., 2020).

Refusals of care can be unimaginably difficult. If caregivers do not know how to handle refusals, they could lead to abuse of the person either through neglect or non-consensual care causing distress or harm (Backhouse et al., 2018; World Health Organisation (WHO), 2020). Person-centred care was demonstrated in our data through both caregiver groups identifying with the personal perspective of the person, changing their schedules, approaches, care activities, and communication to accommodate the person with dementia (Brooker & Latham, 2016). However, it is difficult to understand what personal resources it can take to manage refusals. Most caregivers in our sample were female, sometimes dealing with threatening behaviour from males who may be physically stronger than them. Elder abuse occurs on a continuum (Cooper et al., 2009); in the moment minor issues, or persistent refusals, may push a caregiver over the line between acceptable care and unacceptable care. The challenge is how to draw the line, which may be seen by outsiders as in a different place to where caregivers perceive it to be.

Malpractice within care homes is often portrayed in the media and in research (Moore, 2019). However, although abuse occurs in family settings (Cooper et al., 2009), there is no regulation for family care and daily practices are largely unknown. While obvious criminal acts need to be condemned, the complexity of refusal situations needs to be better appreciated and support provided to prepare and assist caregivers to cope when they arise. Acknowledging the difficulties faced by family carers and care-home staff can be a first step to understanding and valuing their difficult work and offering appropriate support. With greater knowledge, other health and social care practitioners could work closely with both caregiver groups to overcome or circumvent difficulties prior to reaching crisis points or instances of abuse.

### Strengths and limitations

The strength of this research is the focus on two likely care situations (namely, care homes and family homes) allowing learning from both settings. The focus was not on verifying what caused refusals of care, or if refusals were intentional or reactional from the care recipient perspective. Limitations include not knowing how inappropriate strategies are employed, since those people who knowingly use such strategies would not have mentioned them in interviews or volunteered to participate. The sample came from one geographical area of England and comprised of one ethnicity (White British). Research examining this issue in particular ethnic or cultural groups would extend the findings. Teamwork strategies used by family carers alongside paid homecare workers were not addressed in this study.

## Conclusions

Caregiver contexts and relationships with the person with dementia influenced how they managed refusals of care. When facing refusals, the lines between uncompleted care leading to neglect and non-consensual care leading to abuse can be difficult to identify from within the caregiving relationship. Acknowledging these difficulties can be a first step to appropriately valuing caregivers’ work. Health and social care professionals should consider how they can best support family carers and care-home staff to manage difficult personal care interactions. This qualitative study has uncovered rich dynamics between caregivers and care recipients in what can be challenging situations - assisting people who refuse care. Future research should focus on determining the limits and safety factors in this area of practice and the most acceptable ways to provide personal care assistance to people with advanced dementia, for example, by examining real-life personal care interactions.

## Supplemental Material

sj-pdf-1-dem-10.1177_14713012221123578 – Supplemental Material for How do family carers and care-home staff manage refusals when assisting a person with advanced dementia with their personal care? Supplemental Material sj-pdf-1-dem-10.1177_14713012221123578 for How do family carers and care-home staff manage refusals when assisting a person with advanced dementia with their personal care? by Tamara Backhouse, Yun-Hee Jeon, Anne Killett and Eneida Mioshi in Dementia
