# Supplementary material for: How do family carers and care-home staff manage refusals when assisting a person with advanced dementia with their personal care?
Source: Dementia (London). 2022 Sep 2;21(8):2458–75. doi: 10.1177/14713012221123578 (PMC9583289; doi:10.1177/14713012221123578)
Supplement: sj-pdf-1-dem-10.1177_14713012221123578 – Supplemental Material for How do family carers and care-home staff manage refusals when assisting a person with advanced dementia with their personal care? [file sj-pdf-1-dem-10.1177_14713012221123578.pdf]

Supplementary Material

Indicative Interview Topic Guide - Caregivers

Introduction and general chat about topic important to person (pet, career, picture in room, environment/weather) to determine person's ability to take part and build rapport.

Outline parameters of interview – including that we may touch on some impossible dilemmas where there may not be answers.

Supporting the person with dementia (study partner) with personal care

- Describe caregiver role. Enjoy role?
- Talk me through a usual morning care routine (dressing/undressing, bathing/showering, washing, hair care, nail, skin, meds, toileting, oral hygiene, shaving, eating, drinking)
- Differences between evening/morning care
- What like/dislike about looking after person with dementia?
- Refusals? How often? Impact on self/person?
- Are there any aspects that worry you?
- Caregiver knowledge and experiences – burden of role
- Time for care

Strategies Used

- How cope with refusals? If difficult, why?
- You mentioned.... as being difficult to cope with- How cope with it?
- Describe last time you experienced a refusal of care– what did you do?
- How know what to do? How decide?
- How find out about ways to cope with refusals?
- Different strategies for different refusal behaviours? E.gs. How work? Probe....
- Successful ways of coping- how work? Always successful?
- Unsuccessful strategies – why?
- Difficulties with strategies Time/resources?
- What makes it hard to provide care for the person/person with dementia? What could make it easier?
- Tensions – Knowledge/time/experience?
- Use of key words?
- Impact on the caregiver
- Emotion-led care
- Trust (person with dementia – carer; family carer – paid carer)
- Care home staff only: All staff act the same?
- Family carer only: paid carer – intrusion (supportive/negative), continuity, impact; refusals = fear of them not coming
- Teamwork – care home/paid carers/family members

Available Support

- Training
- Advice/information availability
- Do you discuss ways to cope with refusals with anyone?
- Support available? Where? How used?
- Where/how can you access support?
- Is there enough support? Does it help?
- What support would help?
- Resources available? For what?
- Navigating services – work required from carer

Supplementary material

Analysis categories

**Main category**	**Generic categories**	**Subcategories and example data**
**Management of refusals of care**	**Finding the right moment to care** (underpinned by knowing the person)	**Leaving and returning later**<br>*I'd get that done and then out would come the arms again, so I'd go back and sit on the toilet and leave him until he'd got out of his little paddy or whatever you wanted to call it.* (Family Carer 36, Spouse)<br>**Changing the timing of care**<br>*…so, she's got dentures, a full-set, and erm yeah, she, yeah, like I said, she really doesn't like taking them out. So, you have to kind of coax her, get her* ***at the right time****. Erm often it's just before she goes to bed.* (Family Carer 16, Daughter)<br>**Addressing unmet needs first**<br>*…so, talk to them about what's worrying them but then 'would you like a cup of tea or would you like something to eat?' you know kind of like, you know because they might want a cup of tea, they might be you know dehydrated* (Care Home 2, Care Assistant 1)
	**Using specific communication strategies** (underpinned by knowing the person)	**Encouraging / Explaining**<br>*sometimes you can just say "oh come on" and cajole her a bit and just go "come one, it's time to get up" and stuff like that.* (Care Home 3, Care Assistant 1)<br>**Showing emotions**<br>*I've tried being angry or sad or I've tried to cry and just there is no response at all [chuckle]* (Family Carer 8, Daughter)<br>**Being firm**<br>*he doesn't want to do anything, so, so you say "right come on [name] you've got to" [trails off]. [Deep breath] anyway erm [pause] eventually we get his pyjamas off and it is a struggle* (Family Carer 4, Spouse)<br>**Describing the care activity in a minimising way**<br>*I go 'I have* ***just*** *got to do a* ***little*** *freshen up down below is that alright?' and they will either stand up or they hold onto a bar so that I can assist them again, front and back and if they are wearing the pads, I get the pad on, I go* ***'nearly there'*** (Care home 2, Care Assistant 4)
	**Being tactful: simplifying, leaving, or adapting care** (underpinned by knowing the person)	**Providing reduced care**<br>*she doesn't like being out of bed, we have had people here that have had bed days all day and again, it's their choice. As long as it doesn't happen too much, …if you want a bed day, you have bed day. We still try and get them up, …we still try and give them personal care even if they*

		have a nightshirt put back on and then back into bed because then at least they've had some sort of personal care (Care Home 3, Care Assistant 1)<br>**Leaving care uncompleted**<br>*it's easier, just to melt down and say 'okay well if you don't want one [bath] it doesn't matter' you know and I think that's the easiest way because you wind yourself up and it is not that important if he doesn't have a bath, if he wants to be smelly well I can change the sheets next day and give him clean pyjamas you know it doesn't, it isn't life or death (Family Carer 20, Spouse)*<br>**Changing the mode of care**<br>*…I would say, "come on, I'm going to wash your hair today" [reply] "I don't need to wash my hair today!" She'd only wash it once a week …so I bought dry shampoo* (Family Carer 6, Spouse)
	**Having confidence in care** (underpinned by knowing the person)	**Routine**<br>*It's very important to [name], yeah she likes that routine, well that's the routine I've always done with her and that's what she seems to respond to well.* (Care Home 5, Care Assistant 1)<br>**Distraction**<br>*she is yelling the place down, so I started going 'meow meow meow meow' and she stopped and so the other girl said, 'oh I think you're shouting so loud you are upsetting your cat' and so she went 'oh' and then she got her distracted, she got her cat, and she was fine.* (Care Home 2, Care Assistant 3)<br>**Cheerful approach**<br>*if you go in with the right sort of banter and the way you talk, she is pretty good but obviously it is like everyone, you get your good, you get your bad days* (Care Home 2, Care Assistant 4)<br>**Continuing with care**<br>*I'd say, "take your trousers off [name]." "No, I'm going to bed in them." "You can't go to bed in your trousers, look here's your pyjamas, let's put these on, you need to take those off." "No." And then I thought what Dr [name] had told me one time. The odd time shout at him but don't do it often because it won't work if you use it and I just looked at him and I said "[name], you WILL take your trousers off." I've never seen a pair of trousers undone as quick in all my life. … And then I'd feel guilty* (Family Carer 36, Spouse)<br>**Creating time for care**<br>*[Day centre] is a bit hard for me because getting, chivvying [the person living with dementia] along to get ready… takes time… So, what …I need to do, is get up earlier on a Thursday morning because we need to be there at 9am* (Family Carer 41, Friend)
	**Seeking**	**Bringing in others**

	**support from others when safety is at risk** (underpinned by knowing the person)	*At the beginning oh he did not want them [homecare workers]. I had to really really be strong and say, "listen if you want to stay at home, you've got to accept this because I can't cope." And it was difficult at the beginning because he was on double-ups four times a day so you couldn't get the continuity with the carers. But now we've got it down to just mornings and we've just got the two carers, he's fine* (Family Carer 40, Spouse)<br>**Calling on services**<br>*I did have to ask the Doctor for a little bit of help because I was getting to the stage thinking I can't do this anymore, I have been to that stage* (Family Carer 21, Spouse)<br>**Using more than one person**<br>*if there is two of you, one can actually talk to him face to face and the other one is assisting* (Care Home 2, Care Assistant 4)
